# The Benzopyrone Biochanin-A as a reversible, competitive, and selective monoamine oxidase B inhibitor

**DOI:** 10.1186/s12906-016-1525-y

**Published:** 2017-01-10

**Authors:** Najla O. Zarmouh, Suresh K. Eyunni, Karam F. A. Soliman

**Affiliations:** College of Pharmacy and Pharmaceutical Sciences, Florida A&M University, Tallahassee, FL 32307 USA

**Keywords:** Human monoamine oxidase-A, Human monoamine oxidase-B, Benzopyrone, *Psoralea corylifolia* ethanolic extract, Flavonoids, Coumarins, Biochanin-A, Reversible competitive inhibition, Docking studies

## Abstract

**Background:**

Monoamine oxidase-B (MAO-B) inhibitors are widely used in the treatment of Parkinson’s disease. They increase vital monoamine neurotransmitters in the brain. However, there is a need for safer natural reversible MAO inhibitors with MAO-B selectivity. Our previous studies showed that *Psoralea corylifolia* seeds (PCS) extract contains compounds that inhibit monoamine oxidase-B.

**Methods:**

In this study, six of PCS constituents sharing a benzopyrone structure were investigated. The compounds Biochanin-A (BIO-A), isopsoralen, 6-prenylnaringenin, neobavaisoflavone, psoralen, and psoralidin, were tested for their ability to inhibit recombinant human MAO-A and B (*h*MAO-A and *h*MAO-B) isozymes. The ability of these compounds to inhibit MAO-A and MAO-B were compared to that of PCS ethanolic extract (PCSEE) using spectrophotometric assays and confirmed by luminescence assays. The highly potent and selective MAO-B inhibitor, BIO-A, was further investigated for both isozymes reversibility and enzyme kinetics. Molecular docking studies were used to predict the bioactive conformation and molecular interactions of BIO-A with both isozymes.

**Results:**

The data obtained indicate that benzopyrones inhibited *h*MAO-A and *h*MAO-B with different degrees as confirmed with the luminescence assay. BIO-A inhibited *h*MAO-B with high potency and selectivity in the present study (IC_50_ = 0.003 μg/mL) and showing 38-fold more selectivity than PCSEE (*h*MAO-B IC_50_ = 3.03 μg/mL, 17-fold selectivity) without affecting hydrogen peroxide. Furthermore, BIO-A reversibly and competitively inhibited both *h*MAOs with significantly lower inhibitory constant (K_i_) in *h*MAO-B (3.8 nM) than *h*MAO-A (99.3 nM). Our docking studies indicated that the H-bonds and hydrophobic interactions at the *human* MAO-A and MAO-B active sites contributed to the reversibility and selectivity of BIO-A.

**Conclusions:**

The data obtained indicate that BIO-A is a potent, reversible and selective MAO-B inhibitor and may be recommended for further investigation in its possible use in the therapeutic management of Parkinson’s and Alzheimer’s diseases.

## Background

Human monoamine oxidases A and B isozymes (MAO-A and MAO-B) regulate neurotransmitters such as dopamine (DA), norepinephrine (NE), and serotonin (5-HT) [1]. These metabolizing isozymes belong to the family of flavin-containing amine oxidoreductases and are in the mitochondrial membranes of brain neurons and glia, in addition to other peripheral cells. In neurological disorders with depleting neurotransmitters, selective and non-selective MAO inhibitors (MAOIs) are used. The specific MAO-BIs are well-established therapeutics for Parkinson's disease (PD), and currently paving new avenues for Alzheimer disease (AD) patients [2]. Meanwhile, the non-selective and selective inhibitors of MAO-A (MAO-AIs) are used primarily for patients who have not responded to other antidepressants therapy [1]. Some of these therapeutic agents such as moclobemide and pirlindole are currently in use.

MAOIs can exert multiple pharmacological actions by reducing the enzymatically formed hydrogen peroxide (H_2_O_2_) and aldehydes cytotoxic byproducts [3], antiapoptotic effects [4] and increasing DA which indirectly attenuates nitric oxide [5]. These pharmacological properties of MAOIs can provide neuroprotection against oxidative stress and cell death [1], making them effective against neurodegeneration. However, the inevitable rare incidences of the MAOIs side-effects reaction are causing concerns. Nonetheless, the newly emerged class of reversible inhibitors of MAO-A (RIMA), clinically showed almost no cheese effect or need for diet restrictions [6]. Thus, it is important to find reversible inhibitors for effective and safer use emphasized investigations for a new level for MAOs inhibition and MAO inhibitors.

It is of interest to note that some synthesized coumarin derivatives have potent MAO inhibitions [7, 8]. In addition, natural coumarins and flavonoids were found to be beneficial for neurological disorders [9, 10], with potent effects on the synthesis of catecholamines and oxidative stress [11, 12]. Such shared general benzopyrone structure between flavonoids and coumarins emphasize a high potential to inhibit MAO-B. Notably, *Psoralea corylifolia* L. seeds (PCS) (Plant common name: bakuchi, family: Leguminosae) [13] contain a variety of benzopyrone structures as in coumarins and flavonoids derivatives with chromone core structures [14]. We previously reported that PCS ranked one of the most potent plant extracts to inhibit recombinant human (*h*) MAO-B activity among 905 screened extracts [15]. Further, we have shown the significant selectivity of these compounds to MAO-B as compared to MAO-A [16]. Furthermore, the unique constituent of PCS., i.e., bavachinin, showed a reversible *h*MAO inhibition with an *h*MAO-B relative selectivity (RS_B_) [17]. In a previously reported phytochemical screening, the plant seeds were suggested to be a good source for phytomedicines [18]. Indeed, PCS is one of the most popular traditional medicines officially listed in the “Chinese Pharmacopoeia” and has a variety of unique beneficial phytochemical constituents. The PCS seeds have been recently shown to have chemoprotective and antioxidant properties [19]. Some of its investigated phytochemicals showed phytoestrogenic [20], anti-inflammatory [21], and neuroprotective [22] properties. Moreover, the seed’s aqueous extract was found to inhibit DA and NE transporters [23]. Such properties point the need to investigate benzopyrone moiety of the seeds for their MAO inhibitory effects.

**Fig. 1:**
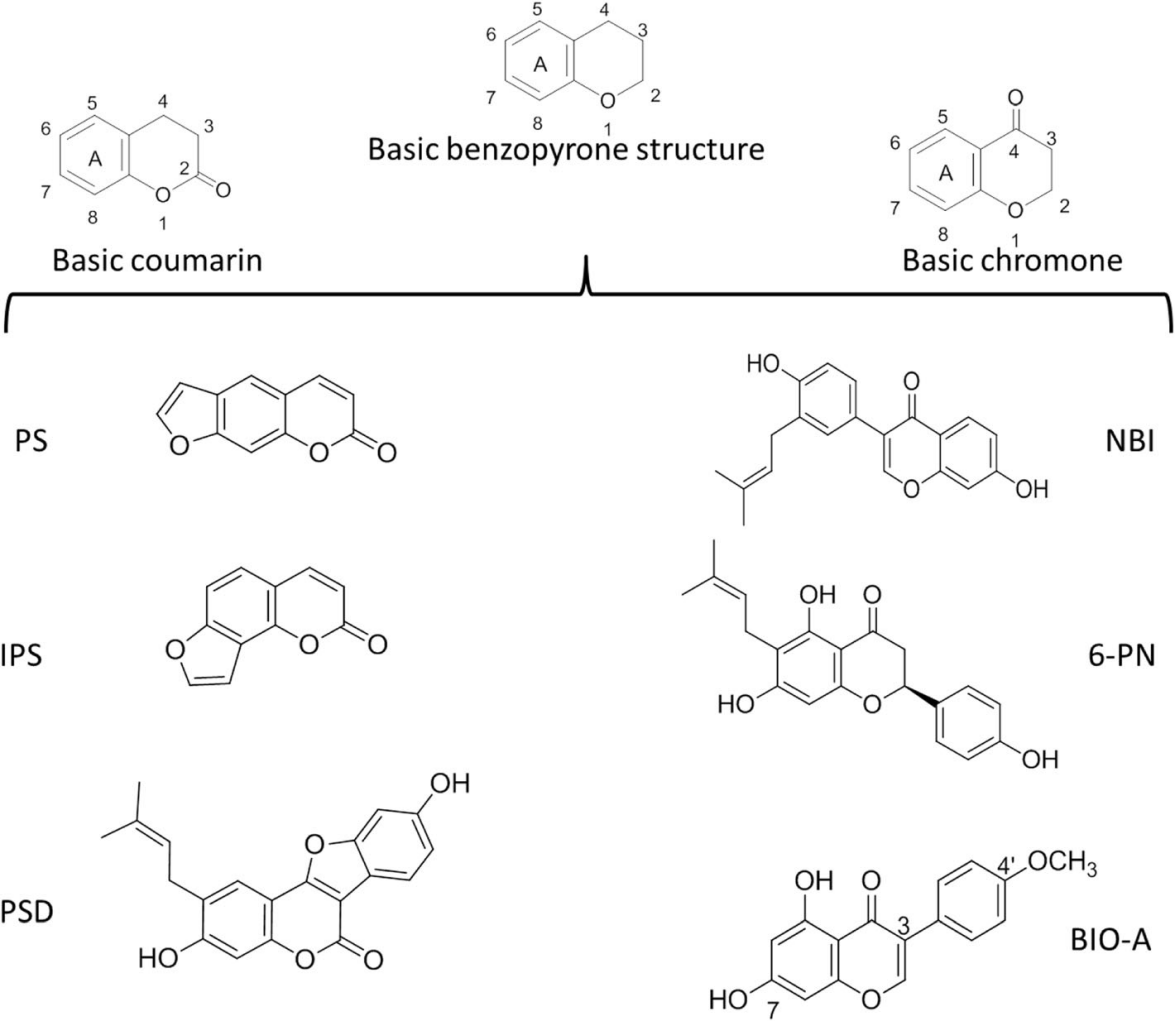
The structures of six *Psoralea corylifolia* seeds (PCS) benzopyrone constituents: Coumarins include psoralen (PS), isopsoralen (IPS), and psoralidin (PSD). Flavonoids of chromone structure include 6-prenylnaringenin (6-PN), neobavaisoflavone (NBI), and biochanin-A (BIO-A)

In the current investigation, we selected six commonly known coumarins and flavonoids that share the benzopyrone structure (Fig. 1.). The selected coumarins included psoralen (PS), isopsoralen (IPS), and psoralidin (PSD). The selected flavonoids studied include 6-prenylnaringenin (6-PN), neobavaisoflavone (NBI), and biochanin-A (BIO-A). These benzopyrones are reported to be in PCS [19, 24]. However, the *h*MAO-A and *h*MAO-B inhibitory effects and selectivities of these compounds remain to be elucidated. The need to find natural selective and reversible *h*MAO-B inhibitors motivated us to evaluate inhibitory activity and selectivity of six of PCS constituents with benzopyrone structures on *human* MAO-A and MAO-B.

## Methods

### Reagents

The tested pure PCS constituents (≥95%) of PS and PSD and the standard selective MAO-AI clorgyline (CLORG) were obtained from Santa Cruz Biotechnology Inc. (Dallas, TX, USA). Hank's Balanced Salt Solution (HBSS), HEPES, benzylamine HCl, IPS, 6-PN, NBI, BIO-A, the selective MAO-BI standard selegiline (Deprenyl®) (DEP), and other chemicals and chromatography supplies were purchased from Sigma-Aldrich (St Louis, MO, USA). Human MAO-A and MAO-B, derived from a recombinant baculovirus infected insect cells, and measured in active units (U) were purchased from Sigma-Aldrich and received in a buffer solution at pH 7.4. The China originated PCS were obtained from East Earth Trade Winds (Redding, CA, USA), and the MAO-Glo™ kits from Promega (Madison, WI, USA).

### PCS extraction

The dry PCS were milled and sieved into 30 g of fine seed powder and stored in a nitrogen-sealed container at 2°C. To prepare PCSEE, we followed the procedure mentioned in our previous study [17]. Briefly, the fine PCS powder was macerated for two days using 99.95% ethanol. The extract was filtered and the powder obtained was subject to 8-10 h Soxhlet reflux extraction at 60° to 70 °C with continuous solvent (ethanol) renewal every 2 h. The combined ethanol extracts were evaporated in a dark hood to obtain an oily non-homogenous PCSEE. All the collected dry crude extracts and prepared PCS ethanolic stock solutions were stored in glass containers at 2 °C until use.

### *h*MAO-A and *h*MAO-B enzymatic activity assays

A slightly modified continuous spectrophotometric assay [25] for measuring enzymatic H_2_O_2_ production was validated before the experiments. In this assay, the peroxidase chromogen reagent of 1 mM of vanillic acid and 500 μM 4-aminoantipyrine, and 4 U purpurogallin/mL of horseradish peroxidase (HRP) type-II in HBSS (pH 7.4) was prepared and validated with H_2_O_2_ standard curve. The linearity of reaction on the instrument was confirmed to reach up to 1.76 nmol per reaction volume with R^2^ of 0.9999. Isozymes were diluted to 0.7 U/mL final concentration by 10 mM HEPES in HBSS (pH 7.4). Substrates final concentrations were optimized to be 0.5 and 3 mM tyramine HCl and benzylamine HCl for *h*MAO-A and *h*MAO-B reactions. The assay was run with and without different standard MAOIs with different selectivities (DEP, CLORG, pirlindole, and rasagiline) and monitored for few hours. Isozymes were found stable within the time of incubation. Stock solvents (ethanol or dimethyl sulfoxide) were kept ≤ 2% the final volume of all assays. IC_50_s were measured at earliest readings with high signal to noise ratios possible were H_2_O_2_ formed < 10% for *h*MAO-A and *h*MAO-B. For the confirmatory luminescence assay, using MAO-Glo™ kit, the protocol was followed to determining the time of incubation at 60 min at RT guided by Valley’s method [26] and the preliminary experiments which we conducted.

### *h*MAO-A and *h*MAO-B spectrophotometric assays

The six benzopyrones of 5 × final concentrations between 0 to 400 μM and PCSEE from 0 to 250 μg/mL of at least ten data points were prepared in HBSS (pH 7.4). The diluted compounds (25 μL) were incubated with 50 μL of 2.5 × the final isozyme concentration (or buffer for blanks) in 96-96-good plates for 40 min at RT. Freshly prepared volumes of 50 μL of 2.5 × substrate and peroxidase chromogen mix (1:1) were added. Absorbance was immediately read at 490 nm by the Bio-Tek μQuant Monochromatic Microplate Spectrophotometer for time zero. H_2_O_2_ production progression for each enzyme was measured every 20 min for the duration of the assay. First read measurements and the blanks background were subtracted from all absorbance values.

### *h*MAO-A and *h*MAO-B luminescence assay

The six benzopyrones, PCSEE, and DEP inhibitory activities of *h*MAO-A and *h*MAO-B isozymes were confirmed using MAO-Glo™ kit luminescence assay. Briefly, in white opaque 96-well microplates, 12.5 μL of 4 × 10 μg/mL final concentrations of each test compound, standard DEP, or PCSEE in reaction buffer of pH 7.4 were added (*n* = 3 per isozyme). A buffer of 12.5 μL was used to make equivalent blank volumes of wells without enzyme. Equivalent amounts of used solvent volumes (ethanol or DMSO) were also added to separate wells. A volume of 25μL of 2 × 0.9 U/mL final concentration of each of freshly thawed *h*MAO-A and *h*MAO-B isozymes were incubated with the test substances at RT. Freshly prepared luciferin derivative substrate volume of 12.5 μL of 4 × 40 and 4 μM the final concentrations were incubated for 60 min with *h*MAO-A and *h*MAO-B and inhibitors, respectively at RT. Reporter luciferase detects reagent of 50 μL was incubated with each reaction for 20-30 min to inhibit the enzyme and produce luminescence. Arbitrary light units (ALU) signal changes were measured by Synergy HTX Multi-Reader from Bio-Tek and converted to percent control change.

### H_2_O_2_ scavenging activity assay

The H_2_O_2_ scavenging activity of PCSEE and BIO-A at their highest used MAO inhibitory concentrations was investigated and compared to the standard scavenger L-ascorbic acid. In 96 well plates, 25 μL of 5 × final concentration of PCSEE (62.5, 125, and 250 μg/mL), BIO-A (6.25, 12.5, and 25 μg/mL), and ascorbic acid (62.5, 125, and 250 μg/mL) in HBSS (7.4) were prepared. A volume of 75 μL of 1.67 × final concentration of 15 μM H_2_O_2_ in HBSS (7.4) was plated in test wells (*n* = 4). Buffer substituted the H_2_O_2_ (*n* = 4) to make blank wells. Immediately, peroxidase chromogen-containing vanillic acid, 4-aminoantipyrine, and HRP type-II were prepared as in the MAO spectrophotometric assay and added as 25 μL per well. The developing color at 490 nm was optimally read within 15 min at RT using Bio-Tek Synergy HTX Multi-Reader absorbance.

### Fluorescent thin-layer chromatography analysis

PCSEE and BIO-A were concurrently run on a silica gel matrix (5 × 10 cm^2^) containing a fluorescent indicator with 254 nm excitation on aluminum foil (Sigma-Aldrich) for thin-layer chromatography (TLC) identification. On replicate TLC matrices, 5 μL of a 1 cm short band of 20 mg/mL PCSEE, and 1 and 5 mM BIO-A (Sigma-Aldrich) ethanol stock solution were directly deposited at 0.5 cm or more from TLC plate lower edge. After ethanol evaporation, TLC plate was slowly placed and developed in a clean glass developing chamber saturated with a miscible solvent system (water: acetone: ethanol of 5: 3: 1 ratio), 0.5 cm deep or less. Plates were taken out after 50-60 min incubation at RT, or when solvent front reached 1 cm from the upper TLC edge (approximately 8.5 cm height). Dried developed TLC plates were then visualized under short (254 nm) and long (366 nm) UV lights. Retention factor (R_*f*_) of BIO-A band and a matching band were quantified.

### *h*MAO-A and *h*MAO-B recovery

*h*MAO-A and *h*MAO-B were tested for their activity recovery after preincubation with BIO-A, utilizing a slightly modified method reported by Legoabe et al [27] and using the continuous spectrophotometric assay [25]. Briefly, in HBSS medium (pH 7.4), BIO-A of 10 × and 100 × IC_50_ and DEP at 10 × IC_50_ were preincubated separately with 70 U/mL *h*MAO isozymes for 40 min at RT. BIO-A of 4 × IC_50_ was concurrently preincubated with 0.7 U/mL. Buffers substituted inhibitors and isozymes for negative controls and blanks, respectively. All reactions were diluted to 20-fold. The 100 × dilution was immediately accomplished by adding 50 μL of the isozyme-inhibitor mixture to 75 μL of related substrates-peroxidase chromogen reagent mix (a 25 μL buffer and the original 50 μL previously used a mixture) in a 96-well plate. By reaching 0.1 × and 1 × IC_50_ BIO-A final concentration, developing color was immediately monitored using Bio-Tek μQuant Spectrophotometer readings at RT.

### Michaelis-Menten kinetics

The effects of BIO-A on Michaelis-Menten kinetics parameters of *h*MAO-A and *h*MAO-B were investigated using the same spectrophotometric assay mentioned above. Standard DEP was concurrently tested to validate the method. Determined DEP kinetics for *h*MAO-A and *h*MAO-B in the conditions of this experiment were determined and used as a standard for the Prism program validation for Inhibitor constant (K_i_) determination of BIO-A. Briefly, seven serially diluted tyramine and benzylamine concentrations were prepared in a range between 0 to 1 mM for *h*MAO-A and 0 to 6 mM for *h*MAO-B. BIO-A for a final concentration of 0, 0.5, 1, and 2 × IC_50_ concentrations were incubated with each isozyme for a final concentration of 0.7 U/mL in a 2:1 ratio (0, 1.7, 3.4 and 6.9 μM for *h*MAO-A, and 0, 0.05, 0.09, 0.19 μM for *h*MAO-B). HBSS (pH7.4) substituted inhibitors and isozymes in the negative controls and the blanks, respectively. After 40 min, 75 μL of the enzyme-BIO-A mixture were added to 50 μL of the related substrate-chromogen reagent mix (1: 1) in 96-well plates. Developing color was monitored at RT using Bio-Tek μQuant Spectrophotometer. Values of maximum velocity (V_max_), Michaelis constant (K_m_), and consequently Ki were determined.

### Molecular docking studies

To understand the possible molecular interactions between BIO-A and isozymes active sites, we used the HYBRID docking method of OEDocking (v 3.0.1) (OpenEye Scientific Software Inc.; Santa Fe, NM, USA) [28]. Both X-ray crystal structures of *human* (h) MAO-A in complex with harmine (PDB ID: 2Z5X) and *human* (h) MAO-B in complex with 2-(2-benzofuranyl)-2-imidazoline (2-BFI) (PDB ID: 2XFN) were downloaded from the RCSB Protein Data Bank (PDB). Both proteins structures were imported into Sybyl -X 1.3 environment of Tripos International (St. Louis, MO) where the protein’s chain-A were extracted and refined using the Biopolymer Structure Preparation Tool. Atom types were corrected, amino acid residues were repaired, active water molecules were retained, and all hydrogen atoms were added to both proteins. The cofactor flavin adenine dinucleotide (FAD) and its covalent linkage to the cysteine residues of hMAO-A (CYS: 406: A), and hMAO-B (CYS: 397: A) were retained during the docking process. To be ready for the docking, the energy of each protein was minimized using the MMFF94 charges with the MMFF94s force fields. For docking the compound of interest (BIO-A), the low energy 3D conformers were generated using OMEGA version 2.4.6 of the same software [28]. The bound standard ligands of 2Z5X and 2XFN were sketched using Sybyl sketch and were re-docked to the crystal structures of the proteins for method validation. The root mean square deviation values between retrieved and re-docked poses of the ligands were less than 2 Å. Top ten poses were examined. BIO-A was then docked as a test ligand to both MAO proteins at their Ligand Binding Domain. The H-bonds formed between BIO-A, and amino acid residues of the top poses were measured.

### Statistical analysis

Statistical analyses were performed using GraphPad Prism program version 6.02 for Windows, GraphPad Prism Software Inc. (San Diego, CA, USA). Data points and parameters were expressed as the mean ± SEM of all data. All parameters were calculated from non-linear regressions with the best R^2^ values. Inhibitory concentrations of 50% of isozyme (IC_50_) were obtained by the interpolation of a normalized variable slope logarithmic curve. RS values of all benzopyrones and PCSEE were calculated from their mean IC_50_s as follows:

$$ \mathrm{R}{\mathrm{S}}_{\mathrm{A}}\kern0.5em =\kern0.5em h\mathrm{M}\mathrm{A}\mathrm{O}\hbox{-} \mathrm{B}\ \mathrm{I}{\mathrm{C}}_{50}/h\mathrm{M}\mathrm{A}\mathrm{O}\hbox{-} \mathrm{A}\ \mathrm{I}{\mathrm{C}}_{50},\ \mathrm{R}{\mathrm{S}}_{\mathrm{B}} = \kern0.75em h\mathrm{M}\mathrm{A}\mathrm{O}\hbox{-} \mathrm{A}\ \mathrm{I}{\mathrm{C}}_{50}/h\mathrm{M}\mathrm{A}\mathrm{O}\hbox{-} \mathrm{B}\ \mathrm{I}{\mathrm{C}}_{50} $$

The V_max_ and K_m_ calculated from Michaelis-Menten kinetics model. K_i_ was calculated according to the competitive behavior model as the best fit and Cheng-Prosuff’s equation (K_i (comp)_ = IC_50_/(1+ [S]/K_m_)). SI of BIO-A was calculated from mean K_i_ values (SI = *h*MAO-A K_i_/*h*MAO-B K_i_). The significance of differences between the different groups was determined using one-way ANOVA followed by Dunnett's multiple comparisons test or two-way ANOVA followed by Sidak's multiple comparisons test.

## Results

### PCS benzopyrones Inhibition of *h*MAO-A and *h*MAO-B

**Fig. 2:**
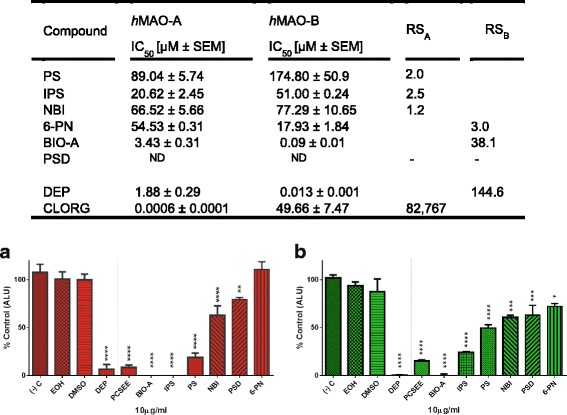
Inhibition potencies and selectivity of *h*MAO-A and *h*MAO-B by PCS benzopyrone constituents. The Table displays IC_50_s of BIO-A, PS, IPS, 6-PN, NBI, and PSD compared with standards deprenyl (DEP) and clorgyline (CLORG), with relative selectivity for isozymes A (RS_A_) and B (RS_B_), using a spectrophotometric assay. **a** *h*MAO-A and (**b**) *h*MAO-B confirmation of inhibition by the six benzopyrones compared to PCSEE, DEP, and solvents of ethanol (EOH) and DMSO using a luminescence assay. IC_50_ ± SEM is interpolated from two sigmoidal curves, and data points were presented as the mean ± SEM percentage isozyme activity of at least two independent experiments of *n* = 3. RS_A_ and RS_B_ are the ratio between high isozyme IC_50_ to the low IC_50_. Significances of differences between groups and control were determined using a one-way ANOVA followed by Dunnett’s multiple comparisons test. * *p* ≤ 0.05, **** *p* < 0.001

To investigate the *h*MAO-A and *h*MAO-B inhibitory effects of the six PCS benzopyrones constituents, we determined their enzymatic H_2_O_2_ IC_50_s using a spectrophotometric assay (Fig. 2 Table) and verified their inhibition by a luminescence assay (Fig. 2 a and b). In both assays, solvents used in compounds stocks showed no significant difference from the controls without solvents. From the first glance at the Table of Fig. 2, benzopyrones of flavonoids and coumarins results clearly indicate an array of *h*MAO-A and *h*MAO-B inhibition potencies with *h*MAO-A and *h*MAO-B IC_50_s less than ~100 and ~200 μM, respectively. Compared with standards CLORG and DEP, *h*MAO-A IC_50_s of the benzopyrones showed BIO-A > IPS > 6-PN > NBI > PS > PSD (no detectable inhibition), with IPS, PS, and NBI having a relative selectivity to inhibit *h*MAO-A (RS_A_). Meanwhile, *h*MAO-B IC_50_s showed BIO-A > 6-PN > IPS > NBI > PS > PSD (partial inhibition, *p* < 0.001), with BIO-A and 6-PN having a relative selectivity in favor to inhibit *h*MAO-B (RS_B_). In this assay, BIO-A was, by far, the most potent and selective *h*MAO-B inhibitor among the six tested benzopyrones.

All the six benzopyrones activities against *h*MAO-A and *h*MAO-B were further confirmed at a fixed concentration using MAO-Glo™ kit luminescence assay, a highly sensitive and an H_2_O_2_-independent technique (Fig. 2 a and b). Standard DEP and PCSEE were used as positive controls. All benzopyrones at 10 μg/mL are differently affected *hMAO*-B *h*MAO-A (*p* ≤ 0.05 to 0.0001), confirming the inhibitory array pattern of the spectrophotometric assay with even higher inhibitory effects. Consistent with our spectrophotometric assay selectivity data, IPS, PS, and NBI, were more effective against *h*MAO-A, and 6-PN was more effective against *hMAO*-B, while BIO-A selectivity was masked by its highly potent effects using luminescence. Thus, the relative selectivity of benzopyrones matching the spectrophotometric assay is an indication that these benzopyrones possess *h*MAO-A and *h*MAO-B inhibitory effects with different selectivities.

### BIO-A and PCSEE Inhibition of *h*MAO-A and *h*MAO-B

**Fig. 3:**
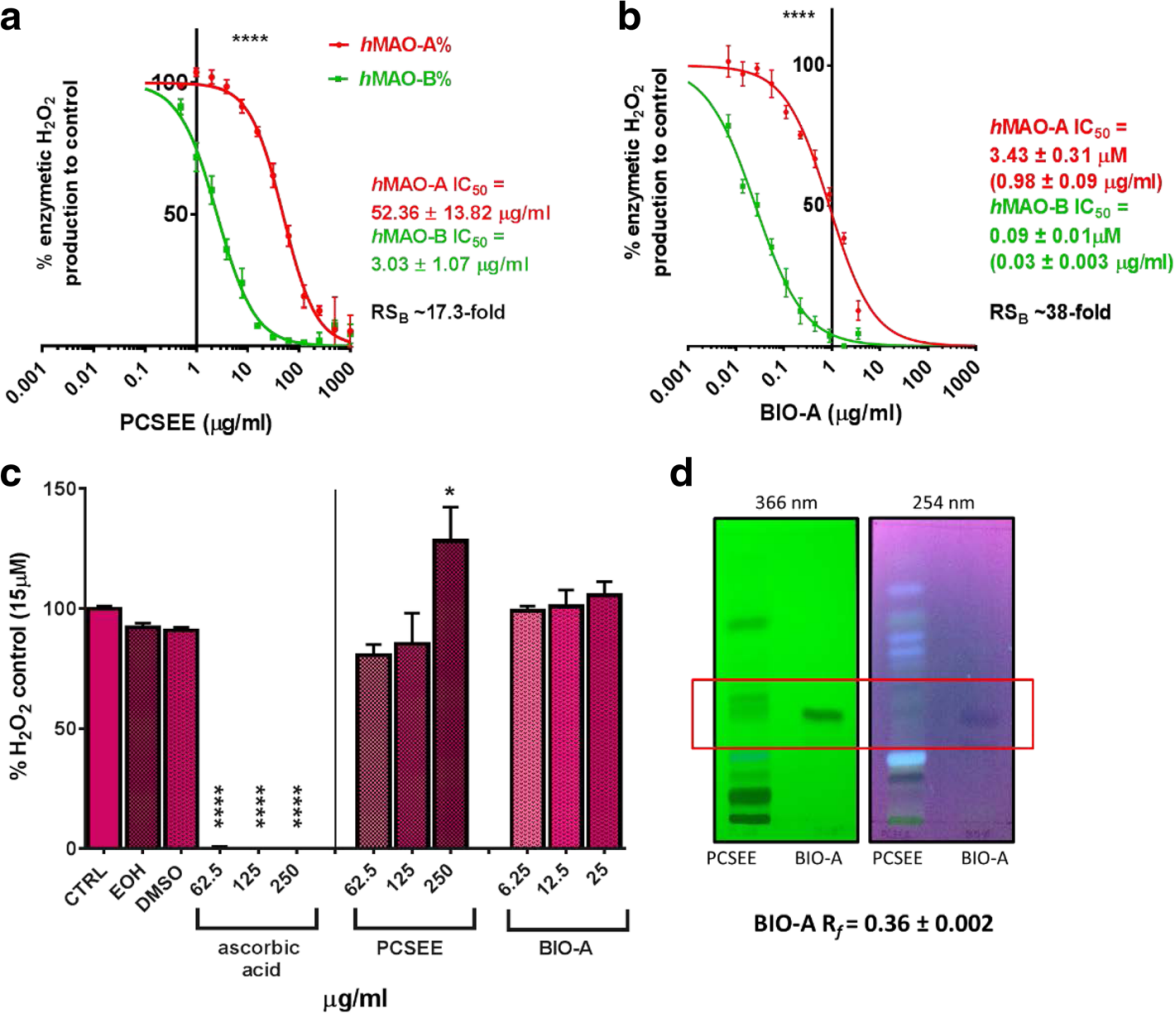
BIO-A and PCSEE selectively inhibited H_2_O_2_ produced by *h*MAO-B more than *h*MAO-A using a spectrophotometric assay without scavenging H_2_O_2_, (**a**) PCSEE inhibited both isozymes from producing H_2_O_2_, and inhibits *h*MAO-B selectively. **b** BIO-A inhibited *h*MAO-A and *h*MAO-B with a higher potency and *h*MAO-B selectivity (RS_B_) than PCSEE. **c** H_2_O_2_ scavenging activity by PCSEE and BIO-A compared to ascorbic acid. **d** Fluorescent TLC plate of PCSEE fingerprint and a BIO-A matching band (R_*f*_ ± SEM) visualized under UV waves. Data points were presented as the mean ± SEM, *n* = 3 (**a**, **b**), 4 (**c**), and 6 (**d**) IC_50_ ± SEM is interpolated from two sigmoidal curves averages. Significance of differences between *h*MAO-A and *h*MAO-B inhibition curves in (**a**) and (**b**) were determined using a two-way ANOVA followed by Sidak’s multiple comparisons test, and between control and treatments in (**c**) using one-way ANOVA followed by Dunnett’s multiple comparisons test; *p ≤ 0.05, ****p < 0.001

To compare the BIO-A inhibitory potencies of *h*MAO-A and *h*MAO-B with PCSEE inhibitory potencies, we elucidated PCSEE effects on both isozymes H_2_O_2_ production using the same spectrophotometric assay (Fig. 3). Ethanol concentrations did not have effects on control isozymes activities. The PCSEE treatments significantly inhibited both isozymes (*p* < 0.0001) (Fig. 3 a). PCSEE showed RS_B_ (17.3-fold) with an IC_50_ of 3.03 μg/mL (*h*MAO-B) compared to 52.36 μg/mL (*h*MAO-A). Nevertheless, the left shifted BIO-A curves compared to PCSEE curves display BIO-A significantly higher potency and RS_B_ than PCSEE (Fig. 3 b).

### BIO-A and PCSEE H_2_O_2_ scavenging activity

H_2_O_2_ scavenging activities were tested to reveal BIO-A and PCSEE direct effects on the enzymatic H_2_O_2_ or other side interactions with the *h*MAOs assays (Fig. 3 c). The ascorbic acid standard showed a complete scavenging activity at all tested concentrations (*p* < 0.001). However, the high tested concentrations of BIO-A and PCSEE did not show any scavenging activities. Moreover, PCSEE increased the H_2_O_2_ signal (*p* ≤ 0.05) which may indicate other autoxidation reactions that occur only at a very high concentration of 250 μg/mL. The results suggest that the inhibition of enzymatic H_2_O_2_ by BIO-A and PCSEE were solely by the mechanism of inhibiting *h*MAO-A and *h*MAO-B, and not by H_2_O_2_ scavenging activities.

### Identification of BIO-A in PCSEE

BIO-A identification was verified in the PCSEE extract using the fluorescent silica gel TLC and our optimized developing system (Fig. 3 d). BIO-A’s non-fluorescent dark band on the TLC plate matched a non-fluorescent dark band in PCSEE fingerprint at both UV excitation waves. At 366 nm, BIO-A band ($Rf$ = 0.36 ± 0.002) was visibly matching one of the PCSEE dark bands ($Rf$ = 0.37 ± 0.002) at similar conditions (*p* > 0.05). At 254 nm with BIO-A increased concentration, BIO-A dark band matched a non-fluorescent dark band in the PCSEE fingerprint developed bands. Thus, in addition to its identification of the used seeds, the chromatographic method supported the reported presence of BIO-A as one of PCS phytochemicals.

### *h*MAO-A and *h*MAO-B modes of inhibition by BIO-A

**Fig. 4:**
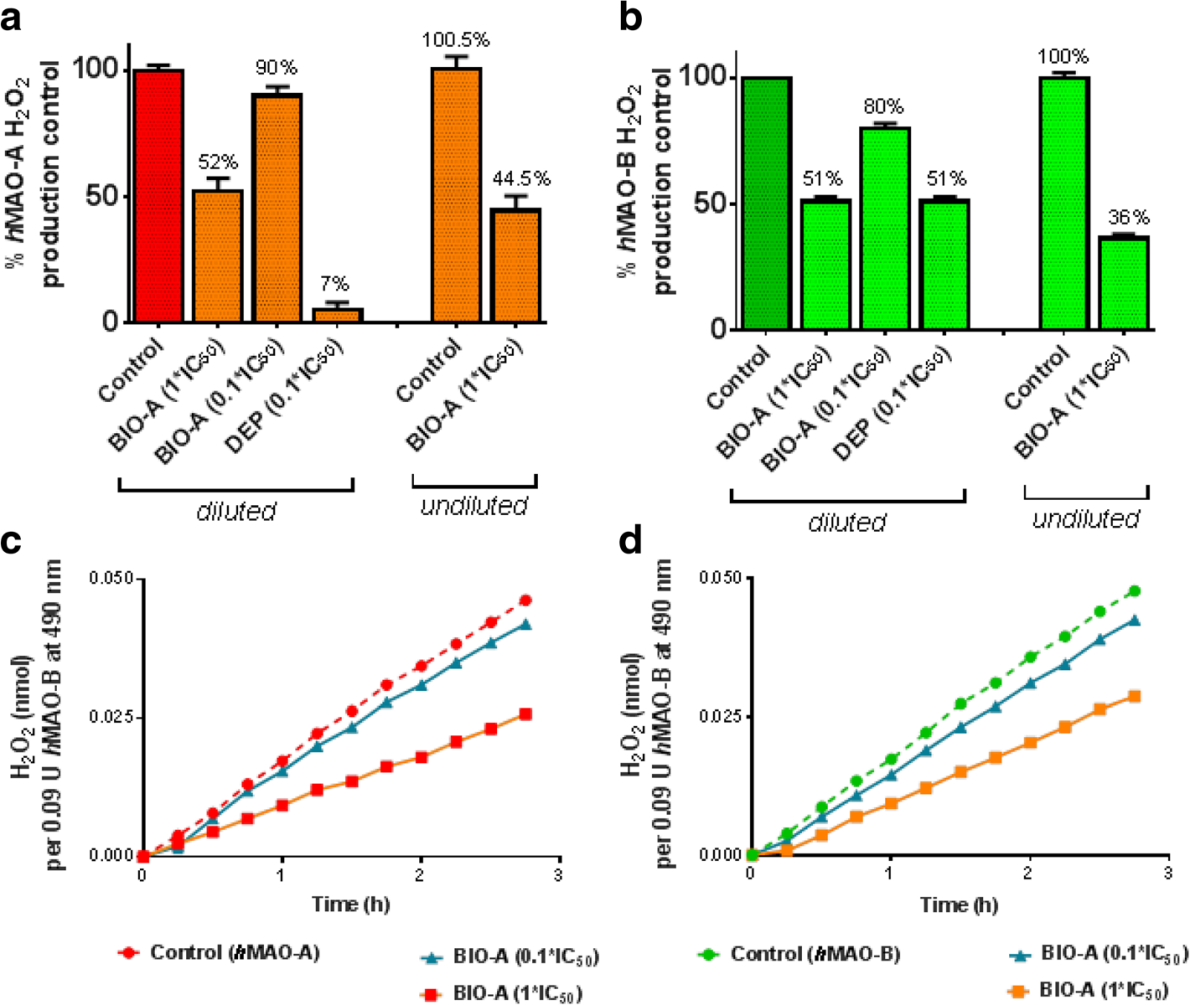
*h*MAOs isozymes recoveries after BIO-A dilution: *h*MAO-A (**a**) and *h*MAO-B (**b**) recovery after preincubation of BIO-A diluted 100-fold to 1 × or 0.1 × IC_50_, compared to diluted standard DEP, and undiluted inhibition data. *h*MAO-A (**c**) and *h*MAO-B (**d**) recoveries were monitored with time at RT. Data points, representing two experiments, and parameters, representing the average of two experiments, at least, were expressed as the mean ± SEM, *n* = 3

**Fig. 5:**
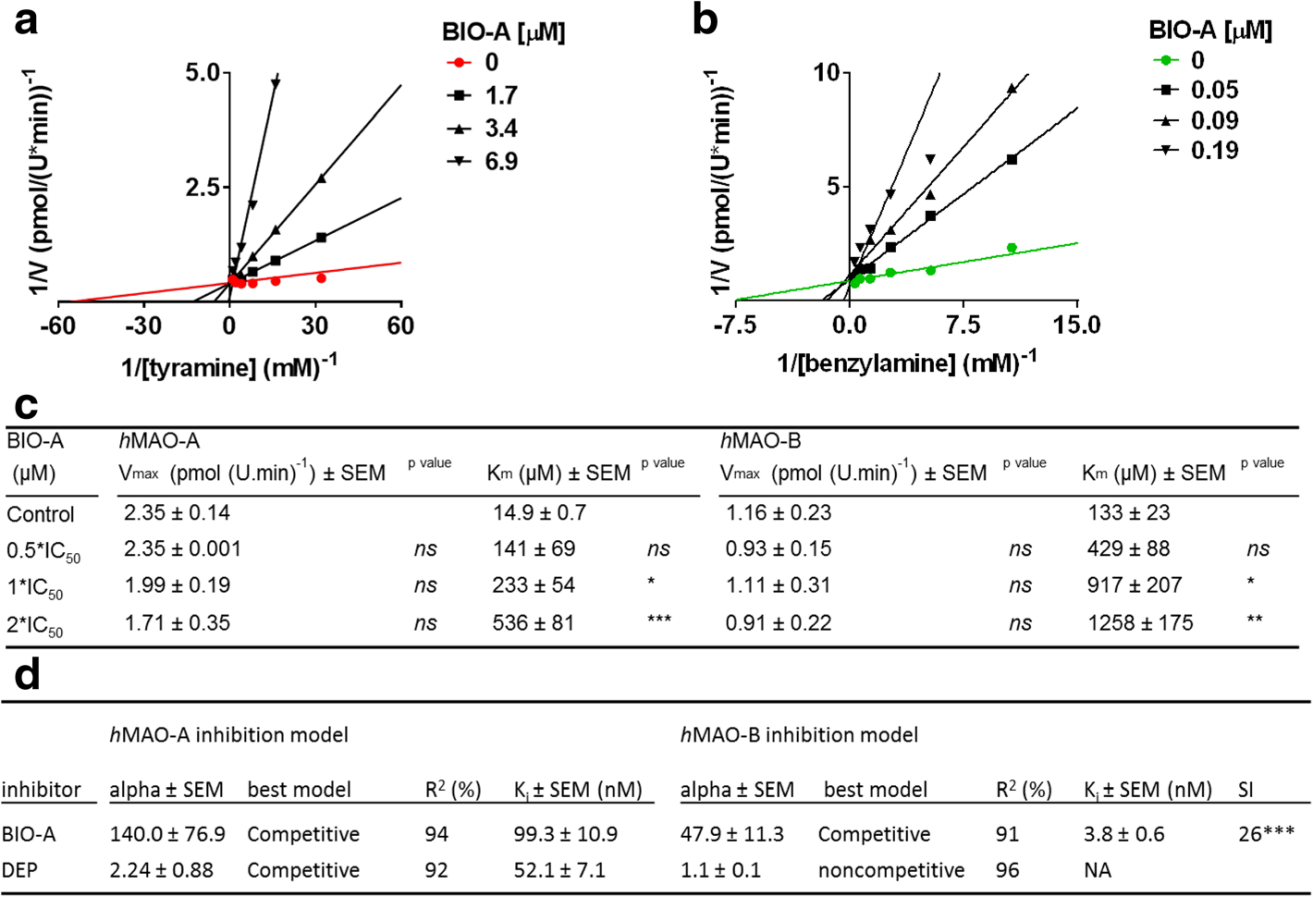
BIO-A effects on Michaelis-Menten Kinetics of *h*MAO-A and *h*MAO-B isozymes: Representative Lineweaver-Burk (LWB) plots of *h*MAO-A (**a**) and *h*MAO-B (**b**) at their initial velocity (V) with increasing tyramine and benzylamine substrates at RT. **c** The effects of BIO-A on the parameters of maximum velocity (V_max_) and Michaelis constant (K_m_) average values in both isozymes. **d** Following *alpha* results, inhibitor constant (K_i_) was calculated using the competitive model and compared to standard DEP: not applicable (NA) for the competitive model. Data points were expressed as the mean ± SEM, *n* = 3, representing two experiments. Parameters data are the average of at least two experiments. The significance of the difference between the control and treatments was determined using a one-way ANOVA followed by Dunnett’s multiple comparisons test, or between two groups using unpaired t test. *ns*: non-significant, *p ≤ 0.05, **p < 0.01, ***p < 0.001

To determine the isozymes mode(s) of inhibition by BIO-A, reversibility of inhibition, Michaelis-Menten parameters changes, K_i_ values for *h*MAO-A and *h*MAO-B, are presented in Figs. 4, and 5, respectively.

### *h*MAO-A and *h*MAO-B reversibility of inhibition by BIO-A

To determine if BIO-A is a reversible *h*MAO-A and *h*MAO-B inhibitor, we tested the recovery of both isozymes from BIO-A inhibition after dilution (Fig. 4 a, and b), with time monitoring (Fig. 4 c and d). Compared to controls, the 0.1 × IC_50_ of standard DEP reduced both isozymes activities to 7% (with *h*MAO-A), and 51% (with *h*MAO-B) instead of 90%, respectively. That is an indication of possible slow, partial, or no recovery of the isozymes from DEP inhibition at the experimental conditions. With *h*MAO-B, repeatedly unexpected observed high signal with DEP could be due to interactions with *h*MAO-B sample contaminants at very elevated levels of drug and enzyme. Conversely, in similar preincubation conditions of BIO-A with *h*MAO-A and *h*MAO-B, dilution allowed a complete or an almost complete recovery of activities. The *h*MAO-A (Fig. 4 a) and *h*MAO-B (Fig. 4 b) catalytic rates with 1 and 0.1 × IC_50_ BIO-A were ~50% and ~90%, respectively. Both isozymes constant recovery with time (Fig. 4 c and d) is a character of a reversible inhibitor, as the activities of recovered enzymes were similar to the enzymes subjected directly to 1or 0.1 × IC_50_ of BIO-A. The obtained results demonstrate evidence that BIO-A is a reversible inhibitor of *h*MAO-A and *h*MAO-B.

### BIO-A-induced changes of *h*MAO-A and *h*MAO-B Michaelis-Menten parameters

To determine the mode of BIO-A reversible inhibition on both MAOs, we investigated BIO-A effects on Michaelis-Menten kinetics of *h*MAO-A and *h*MAO-B at their initial velocities (Fig. 5). In Fig. 5 a and b, Lineweaver-Burk plot linear regressions for the BIO-A increased IC_50_ fold co-intersected at the Y-axis indicating a competitive mode of inhibition in both isozymes. Compared to control in Table of Fig. 5 c, BIO-A showed no significant change in *h*MAO-A V_max_ while K_m_ values significantly (*p* < 0.001) geometrically doubled (9.54, 19.66, 40.38-fold, respectively) with increasing BIO-A IC_50_s fold of concentrations. Similarly, *h*MAO-B V_max_ showed no significant changes while benzylamine K_m_ values were significantly (*p* < 0.01) nearly linearly increased (3.33-, 6.76-, 10.18- fold respectively) with the BIO-A doubled IC_50_s. The obtained data indicate the competitiveness of BIO-A to inhibit both isozymes.

### BIO-A K_i_ and SI determination

To confirm competitiveness of BIO-A for *h*MAO-A and *h*MAO-B inhibitions, and consequently determine BIO-A K_i_ and its *h*MAO-B selectivity index (SI), we analyzed Michaelis-Menten data by best-fit enzyme inhibitory models using GraphPad Prism. (Fig. 5 d; Table). The mixed enzyme inhibition model for standard DEP indicated a competitive inhibitory mode for *h*MAO-A (alpha > 1) and a non-competitive mode for *h*MAO-B inhibition (alpha ~1). Meanwhile, the same model supported that BIO-A impedes substrate-binding affinity to both isozymes (alpha > > 1). Thus, the analysis confirms BIO-competitiveness for both isozymes and rejects the non-competitive, uncompetitive behavior. Consequently, BIO-A K_i_ values showed that BIO-A SI has a tight affinity to bind to *h*MAO-B more than *h*MAO-A (26-fold; *p* < 0.001).

### Molecular docking of BIO-A

**Fig. 6:**
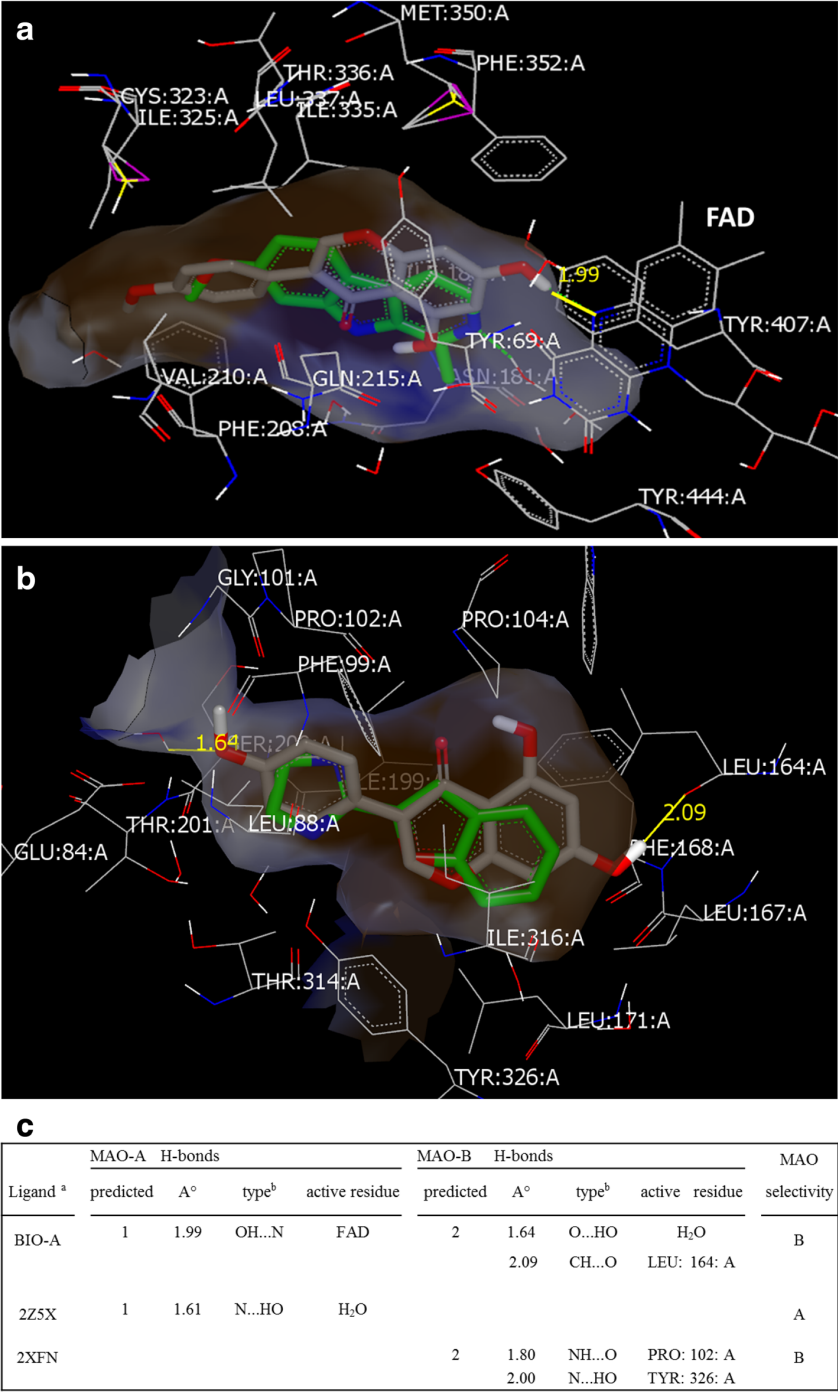
BIO-A docking orientations within *human* MAO-A and MAO-B crystal structure active sites (BIO-A is a gray molecule, active sites: the neutral zones are gray, the lipophilic zones are brown, and the hydrophilic zones are blue in color): (**a**) within MAO-A with 2Z5X_ligand standard (in *green*), and (**b**) within MAO-B with 2XFN_ligand standard (in *green*). In table (**c**), predicted BIO-A H-bonds numbers, distances (Å), type of H-bonds between BIO-A (on left) and residue (on right) (^b^), and involved amino acid residues are compared to the two standard ligands docked within the same site (^a^). Key: LEU: leucine, PRO: proline, TYR: tyrosine at the A chain of MAO-B

To recognize the molecular level of the interactions of BIO-A with the amino acids in the active site involved in its MAOs reversible and competitive inhibitions; we conducted a molecular docking study on both isozymes (Fig. 6). BIO-A was well docked to the same *human* MAO-A and *h*MAO-B crystal structure active sites at which 2Z5X and 2XFN interacted, respectively (Fig. 6). In the MAO-A compact active site cavity, BIO-A matched the 2Z5X pose with its C3-phenyl ring and the chromone core structure embedded in the hydrophobic zone of the entrance site (Fig. 6 a, Brown zone). Also, BIO-A was predicted to be a donor for a relatively tight H-bond (1.99 Å) formed between the hydrogen of its C7-OH group and the nitrogen FAD of the enzyme (Fig. 6 c Table). Therefore, the reversible H-bond with FAD and the hydrophobic interactions at the entrance cavity may contribute to BIO-A reversible competitive behavior in effectively inhibiting *human* MAO-A.

Nonetheless, BIO-A showed stronger interactions at the active site of MAO-B than MAO-A. All of the BIO-A i.e., the lipophilic chromone core structure, the C3-phenyl ring, and possibly the methyl group of C4’ positions, were completely embedded in the MAO-B active site lipophilic zones, away from the FAD (Fig. 6 b, Brown zone). Notably, BIO-A was predicted to be a donor and an acceptor for two distant H-bond. One at the entrance cavity between the oxygen acceptor of its C4’-OCH_3_ group and the active water molecule, and the other, is closer to the substrate cavity between the carbon donor of its C7-OH and the Leucine 164 (Fig. 6 c Table). The BIO-A H-bond distances were close to the 2XFN H-bond distances. The predicted reversible hydrophobic interactions and distant H-bonds formations might consistently reflect the high affinity, reversibility, and competitiveness extent of BIO-A towards its MAO-B mode of inhibition.

## Discussion

As part of our search for safe, natural MAO-B inhibitors, and guided by selecting benzopyrone structures, we focused our efforts to investigate compounds that could be responsible for the plant PCS selective MAO-B inhibition. Furthermore, we characterized the action of the most potent and selective inhibitor. In the current investigation, we tested PCS as well as six of its benzopyrone constituents for their *h*MAO-A and *h*MAO-B inhibitions. The PCS isoflavone constituent BIO-A was found to be the most potent and selective *h*MAO-B inhibitor in this study. BIO-A was also more potent and selective than PCSEE. The presence of BIO-A in PCSEE was verified, and their inhibition of MAOs did not involve H_2_O_2_ scavenging activity. Furthermore, our results indicated that BIO-A is a reversible and competitive *h*MAOI with an SI to inhibit *h*MAO-B. In molecular docking experiments, BIO-A selective activity was accompanied with two reversible H-bonds and three hydrophobic interactions with the human MAO-B active site, which were more than the reversible interactions formed with the MAO-A active site.

The results obtained showed that BIO-A (5, 7-dihydroxy-4'-methoxyisoflavone) is the most potent *h*MAO-A and *h*MAO-B inhibitor with the highest MAO-B selectivity out of the tested benzopyrones and PCSEE (Figs. 2 and 3). The obtained PCSEE results are consistent with our previous reports on the potency and selectivity using spectrophotometric and fluorescence assays with different extraction methods [15, 16]. We verified the presence of BIO-A in our used PCSEE to confirm previously reported 0.063 ± 0.003% w/w of BIO-A in the dried PCS [24]. BIO-A IC_50_ and RS results on both isozymes were consistent with the more reflective parameter of its binding affinity, K_i_; that was higher in *h*MAO-B than hMAO-A (Fig. 5). Notably, BIO-A at a very high concentration becomes less selective due to the structure similarities of both isozymes (Figs. 2 and 3). Similarly, standards pirlindole, DEP, and RAS become non-selective when their concentrations are highly increased.

The obtained results indicate the non-selectivity of the studied coumarins as MAOIs. These results are supported by previous reports which indicate PCS total furocoumarins had antidepressant effects in mice and showed in vitro *rat* MAOs inhibitory effects [29], and another study reported PS and IPS inhibitory effects of rat MAOs [30]. Additionally, we used luminescence assay for confirmation of spectrophotometric assay isozymes effective inhibitions; the luminescence assay is independent of measuring H_2_O_2_ with a lower representation of the biological reactions. The varied inhibitory potencies may differ with the enzyme source and methods used as it was previously reported with other MAO-BIs [31].

BIO-A structure and the tested benzopyrones are absent of any reactive terminal amino groups as in DEP or phenelzine that can covalently bind at the MAO active site. Our docking evaluation indicated that the non-covalent molecular interactions were underlying BIO-A competitiveness for *h*MAO-A and *h*MAO-B (Fig. 5), and thus, supported our biochemical data. In the human MAO-B, BIO-A contained two H-bonds comprising the acceptor and the donor of the C7-OH and the C4’-OCH_3_ groups, respectively (Fig. 1). Previous docking studies indicated that flavonoids H-bond donors or acceptors complemented with lipophilic interactions are candidates to modulate both *rat* MAOs activities [32]. Additionally, two predicted H-bonds at *h*MAO-B in our previous reports of flavonoids bavachinin and genistein were accompanied with competitive *h*MAO-B inhibitory effects with higher *h*MAO-B affinity than MAO-A [17, 33]. Thus, the two H-bond acceptors with the three hydrophobic groups in BIO-A held the best features of the MAO-BI flavonoid pharmacophore more than MAO-A. These consistent results point out H-bonds as a possible critical factor for the human MAO-B affinity stabilization and consequently a better flavonoid inhibitory activity. In MAO-A however, the prediction of a single but crucial H-bond with FAD may explain BIO-A lower affinity, potency, and higher competitiveness to inhibit MAO-A than B.

In addition to our results in *h*MAO-A and *h*MAO-B inhibitions, BIO-A was reported to have several multiple pharmacological functions. BIO-A was reported to be neuroprotective in *in-vitro* and in vivo studies through its multimechanistic antiinflammatory, antioxidant, and phytoestrogenic properties. BIO-A inhibited lipopolysaccharide (LPS)-induced dopaminergic cell damage in rats [34], and LPS-induced activation of microglia [35], protected from the glutamate- and Aβ- induced cytotoxicity in neuropathological rat models [36, 37], and the glutamate-induced cytotoxicity in *human* cortical neurons [38].

More importantly, BIO-A ameliorated age- and drug-induced cognitive and behavioral dysfunctions in mice model [39], improved the neuronal viability, vascular functions, and memory [40], and promoted the recovery of peripheral nerve injuries [41] in rat models. It also reduced acetylcholinesterase in dementia mouse [40] and rat [39] models. Such reports indicate that BIO-A can provide neuroprotective effects in different animal models and provide evidence that BIO-A can cross the blood-brain barrier. From another perspective, BIO-A was reported of being metabolized by 4’-O-demethylation by P450 isoforms mainly CYP1A2 to genistein [42, 43]. Moreover, genistein was also reported to be metabolized back to BIO-A by 4’-O-methylation by rat liver enzymes [44]. Furthermore, in our recent investigations, genistein was found to be an active MAOI [33]. BIO-A with its metabolite genistein shared hepatoprotective [45], antimicrobial [46], and anticancer activities [44]. Having BIO-A and genistein to be human MAOIs, the two analogs mutual presence is highly possible in the blood, and hence, their possible activity in the brain against neurodegeneration.

The present kinetics studies indicate that BIO-A can be a safe and effective MAO-BI. The relatively superior selectivity of BIO-A can eliminate oxidative stress produced by both H_2_O_2_ and toxic aldehydes, which are generated by *h*MAO-B. The reversibility and competitiveness of BIO-A for *h*MAOs inhibitions can also eliminate the classical side effects related to MAOIs irreversibilities such as the cheese effect, psychosis, withdrawal and possible drug interactions. Consequently, BIO-A may safely reduce the aging brain oxidative stress by introducing a new mechanism for reversibly inhibiting both *human* MAOs with MAO-B inhibitory selectivity. There are other natural products with MAO-B inhibition proposed for neuroprotection [47–49], and there is a need to disclose their value in PD and AD patients.

## Conclusions

It was concluded from the current study that BIO-A as a PCS benzopyrone constituent was found to be the most potent and selective *human* MAO-B inhibitor among the studied benzopyrones. BIO-A, and the variable *h*MAO-A and *h*MAO-B inhibitions of benzopyrones may partially explain the PCSEE MAO-B inhibition potency and selectivity. The results also indicated that BIO-A is a reversible and competitive MAOI with a high selectivity index and high affinity to inhibit MAO-B. The predicted interactions of BIO-A with the active site amino acids involve reversible H-bonds and hydrophobic interactions instead of irreversible covalent adducts. This combined mechanism of MAO-B selectivity and reversibility may provide a greater safety margin and prevent the classical MAOIs side effects and following drug restrictions. Supported by previous reports, our data highlights BIO-A potentials to be used for the management of age-related neurodegenerative diseases such as PD, and AD.

## Abbreviations

6-PN: 6-prenylnaringenin
AD: Alzheimer's disease
BIO-A: Biochanin A
CLOG: Clorgyline
DA: Dopamine
DEP: Selegiline (Deprenyl®)
FAD: Flavin adenine dinucleotide cofactor
H_2_O_2_: Hydrogen peroxide
HBSS: Hank's balanced salt solution
hMAO: Human MAO
*h*MAO: *Recombinant human* MAO
*h*MAO-BIs: *Recombinant human* MAO-B inhibitors
HRP: Horseradish peroxidase
IPS: Isopsoralen
MAO: Monoamine oxidase
NBI: Neobavaisoflavone
NE: Norepinephrine
PCS: *Psoralea corylifolia* L. seeds
PCSEE: PCS ethanolic extract
PD: Parkinson's disease
PS: Psoralen
PSD: Psoralidin
R_*f*_: Retention factor
RIMA: Reversible inhibitors of MAO-A
RS: Relative selectivity
RS_A_: RS in favor to inhibit *h*MAO-A
RS_B_: RS in favor to inhibit *h*MAO-B
SI: *h*MAO-B selectivity index
TLC: Thin-layer chromatography
